# The incidence of lung cancer in Northern Ireland: 1991–1992: A comparative study

**DOI:** 10.1007/s11845-021-02783-0

**Published:** 2021-10-16

**Authors:** Pauline Wilkinson, Joe MacMahon, Gilbert MacKenzie

**Affiliations:** 1 King’s College Hospital, London, UK; 2 The Belfast City Hospital, Belfast, Northern Ireland, UK; 3 Centre of Biostatistics, University of Limerick, Limerick, Ireland

**Keywords:** Cancer registration, Incidence, Lung cancer, Regional comparisons, Statistical modelling

## Abstract

**Introduction:**

Lung cancer is the leading cause of cancer deaths in many Western countries, but its incidence has never been studied in Northern Ireland.

**Aims:**

Accordingly, the present study was mounted to determine, for the first time, the incidence of the condition in Northern Ireland and to compare the findings with other regions in the British Isles.

**Methods:**

A notification study of the incidence of lung cancer (ICD 162) was conducted in Northern Ireland during 1991/1992. Notifications from 6 sources were computerised and linked. Incident cases were identified and analysed in relation to Age, Sex and Geographical region—Northern Ireland, England and Wales, Scotland and the Republic of Ireland.

**Results:**

Some 900 incident cases of lung cancer were identified. The incidence rate per 100,000 population was found to be 57.04. Mortality underestimated incidence by 12.5%. ($p<0.05$). The male to female incidence ratio was 2.1: 1, and this ratio was similar in other regions, except Scotland, where the ratio was 1.7:1. The null hypothesis of a common incidence distribution across regions was formally rejected. A variety of models were fitted and a model in which the log-odds on incidence was a quadratic function of age fitted most of the regional data.

**Conclusions:**

Northern Ireland had the lowest incidence of lung cancer in the UK, but its overall rate was still 40% higher than that observed in the Republic of Ireland which had the lowest rate in the British Isles. Across regions, the pattern of incidence by age and sex was complicated, but a linear logistic model fitted all of the Irish data and the female data in Scotland, satisfactorily.

## Introduction

Lung cancer is the leading cause of cancer deaths in many Western countries. It has become the major cause of cancer deaths in males and is only superseded by breast cancer in women. Even this pattern has altered in some countries with lung cancer overtaking breast cancer as the leading cause of cancer deaths in women, for example in Scotland [1, 2].

Differences in the incidence of lung cancer have been reported throughout the world: Scotland, the USA and New Zealand (Maoris) having high rates and India, Israel, Singapore and Thailand having low rates [3–6]. Of particular note is the rapidly increasing incidence within developing and Eastern European countries. As with mortality, differences in incidence have been reported between the sexes—particularly the changing rates among women [7–9]. Such geographical and sex differences may be accounted for, in part, by differing patterns of smoking and tobacco consumption [10–14].

In Northern Ireland, population data pertaining to the incidence of lung cancer have not previously been published. When the study was started (1991), only three papers had dealt with the characteristics of the disease in Northern Ireland [15–17] and, of these, two had focused on mortality [15, 16], while the third [17] dealt with survival in a surgical setting. The lack of interest in incidence, *per se*, is rather surprising, but, may be attributable to the absence of cancer registration facilities in the Province. A properly constituted population-based cancer registration system^1^ was only established in 1994 [18]. Prior to that date, the notification system in use was estimated to be approximately 60% complete in relation to lung cancer registration [19]. The absence of accurate incidence data hampers national and international comparisons and limits planning.

As in other regions of the UK, mortality data are routinely available from the annual reports of the Registrar General (NI). The usefulness of such data is, of course, limited [20–23] and any orthodox approach to the monitoring and control of lung cancer requires a detailed knowledge of the incidence distribution. Accordingly, a comprehensive epidemiological study was set up to measure, *inter alia*, the incidence of lung cancer in Northern Ireland during 1991/1992 and to compare the findings with other regions of the British Isles.

## Methods

These have been described in greater detail elsewhere [24]. Briefly:

### Diagnosis of lung cancer

Diagnosis was based upon existing histological, radiological and clinical evidence which was considered by one of us (PW). Where histological or cytological evidence was lacking, the diagnosis was made by PW based upon available investigations and the clinical course of the illness. In this way, only cases of primary lung cancer (ICD 162, 9th revision) were included in the study.

### Definition of incidence

An incident case was defined to be a person of any age resident within Northern Ireland who was newly diagnosed as having primary lung cancer (ICD 162, 9th revision) during the one year period 1/10/1991 to 30/9/1992. For example, as framed, the definition specifically excludes cases of mesothelioma (ICD 163) or secondary lung cancer.

### Sources of ascertainment

Incident cases were identified during the study period using a multi-source notification scheme. Notification sources included: general practitioners, hospital physicians and surgeons, radiotherapists, pathology laboratories, the office of the Registrar General (NI) and the NI Cancer Notification Scheme.

A simple notification form was designed and circulated to hospital and community-based Physicians and Surgeons at regular intervals together with reminding letters. These forms were completed by the notifying Physicians or Surgeons and returned to the researcher. In addition the thoracic Surgeons provided copies of discharge letters for in-patient, out-patient procedures and admissions. Separate arrangements were made with the Radiotherapy department, Pathology laboratories, Registrar General’s Office and the NI Cancer Notification Scheme enabling them to notify cases routinely. Furthermore, PW regularly monitored the notifying agencies and inspected log-books, computerised listings and other records in individual hospitals and units to ensure that cases had not been inadvertently overlooked.

### Multi-source notifications and incidence

Notifications from individual agencies were computerised in the Department of Epidemiology and Public Health, The Queen’s University of Belfast, and linked using the patient’s name, sex and date of birth. A file of potentially incident cases of lung cancer was produced and up-dated regularly throughout the study period. Following notification, PW reviewed the hospital notes and general practitioner notes (if necessary). For example, even when a notification was based on a single source (e.g., death certificate data) the patient’s hospital notes were retrieved (via a master patient index) to determine whether the case fulfilled the study criteria, and, if so, to abstract other pertinent clinical data. This method was applied for all cases regardless of whether they were notified from single or multiple sources. In this way, the number of incident cases of primary lung cancer occurring in Northern Ireland during the study period, and the pattern of notification among the different sources, was ascertained and verified.

### Comparative data

Population and mortality data for lung cancer (ICD 162) were obtained from the Registrars General of Northern Ireland [25, 26] (1991/1992) and Scotland (1991/1992), respectively. For Scotland, incidence data were obtained from the Scottish Cancer Registry, whereas the Office of National Statistics provided incidence and mortality data for England and Wales as did the National Cancer Registry in the Republic of Ireland (personal communications). In general, the data selected for comparison were the latest (population and matching incidence) available at the time of writing.

### Statistical methods

Standard statistical methods appropriate to the analysis of incidence rates were employed. In particular comparisons, it was found convenient to model the probability of incidence, $\theta$, by the linear logistic function [27], whence the natural logarithm of the ‘odds’ on incidence is given by:

1 $$\log {\theta /(1-\theta )} = \alpha + \beta({\text{Age}}) + \gamma({\text{Sex}}) + \delta({\text{Region}})$$

where $\alpha ,\beta ,\gamma$ and $\delta$ are unknown parameters describing dependence on the three factors studied. The generalised linear interactive modelling package, GLIM [28], was used to fit various forms of model (1) to the incidence data considered later.

In addition, age-standardised rates were calculated using the direct method. The 5% level of statistical significance, which should be regarded as a nominal reporting level, was used in tests of statistical hypotheses and in the construction of confidence intervals (CIs).

## Results

### Ascertainment

During the study period 3814 notification forms were received relating to 1818 individual possibly incident cases of lung cancer. However, of these 1818, the following were excluded: 283 (15.6%) with the wrong diagnosis (for example, no proven malignancy, secondary lung cancer, lymphoma), 605 (33.3%) with a diagnosis outside the study period, 12 (0.7%) who were resident outside Northern Ireland and 18 (1.0%) who could not be traced. In all, 918 (50.5%) cases were excluded leaving 900 incident cases of primary lung cancer for analysis.

Overall, a total of 2739 notifications were received for these 900 incident cases (a ratio of 3:1). Table 1, which groups the sources into 4 major categories S01–S04, illustrates the value of the multi-source notification approach adopted in the study. For example, a single source study based on the NI Cancer Notification System (S04) would have identified only 361 cases, 40.1% of the true incidence, compared with a maximal 676 cases, 75.1%, using only GPs, hospital physicians and other specialists (SO1). No single source studied was sufficiently complete to have been used for serious epidemiological purposes. Moreover, a study employing 3 sources (S01 and S02 and S04) but ignoring death certification data (S03) would have missed 115 cases, 12.8% of the true incidence. Remarkably, only 137 cases (15.2%) were known to all 4 sources during the course of the study.

**Table 1:** Incidence of lung cancer in NI (1991/1992), established by multi-source ascertainment

Pattern	Sources				Frequency	
No.	S01	S02	S03	S04	Number	%
1	+	−	−	−	59	6.6
2	−	+	−	−	22	2.4
3	−	−	+	−	115	12.8
4	−	−	−	+	7	0.8
5	+	+	−	−	101	12.8
6	+	−	+	−	85	9.4
7	+	−	−	+	33	3.7
8	−	+	+	−	38	4.2
9	−	+	−	+	4	0.4
10	−	−	+	+	30	3.3
11	+	+	+	−	119	13.2
12	+	+	−	+	74	8.2
13	+	−	+	+	68	7.5
14	−	+	+	+	8	0.9
15	+	+	+	+	137	15.2
Total					900	(100)

NB: Key, + = Known to source, − = Not known to source

Sources: S01 = General practitioners Hospital physicians and surgeons (specialist/non specialist), Radiotherapists. SO2 = Pathology laboratories. SO3 = Death certification. SO4 = NI Cancer Notification System

### NI incidence

Of the 900 incident cases, 601 were male and 299 were female. The 1991 census for Northern Ireland enumerated the population as 1,577,834 persons (769,057 males and 808,777 females). Based on these figures, the incidence of lung cancer in Northern Ireland during the study year was 57.04 cases per 100,000 population (95% CI = 53.24, 60.84). The corresponding rates for males and females were 78.15 per 100,000 (95% CI = 71.78, 84.52) and 36.97 (95% CI = 32.69, 41.25), respectively. Overall, the male to female incidence ratio was 2.1:1.

**Fig. 1:**
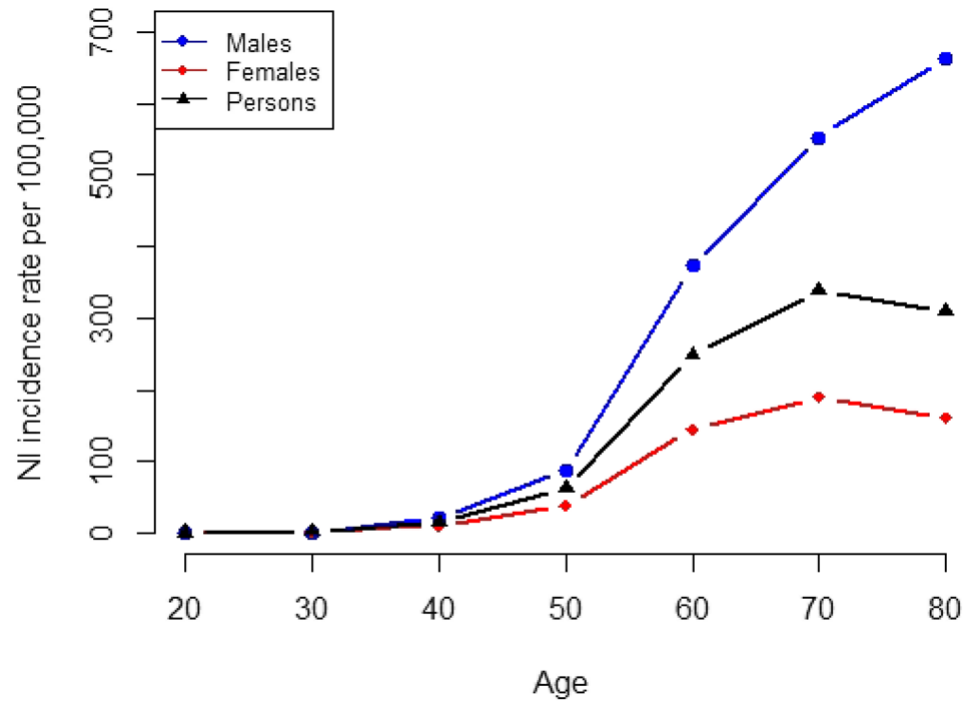
Northern Ireland incidence rates (per 100,000 population) for cancer of the lung (1991–1992) by age (20+ years), sex and persons

Since mortality has been used consistently as a surrogate measure of incidence in Northern Ireland, it is interesting to quantify, for first time, the resulting bias. In 1991, 788 deaths from lung cancer (ICD 162) were reported in Northern Ireland, yielding a mortality rate of 49.94 per 100,000 population. This figure under-estimates the true incidence rate by 12.45% and is computed as 100 $\times$ (57.04–49.94)/57.04). Since the mortality rate falls outside the 95% CI for the true incidence rate, we conclude that the incidence of lung cancer in NI is statistically significantly higher than the mortality. The 1991 sex-specific mortality rates were 73.60 and 27.44 for males and females and these figures underestimate the corresponding incidence rates by 5.82% and 25.78%, respectively. The underestimate for females is rather dramatic and highly statistically significant. A comparison with the mortality rates in 1992 reveals a similar pattern: the overall mortality rate under-estimates the true incidence rate by 16.02%; for males the underestimate is 16.68% and for females the underestimate is 14.93%. All of the mortality rates lie outside the 95%CIs for the corresponding true incidence rates.

Table 2 (a) shows the observed incidence rates (per 100,000) for Northern Ireland by age (10 year groups) and sex. Clear differences in incidence exist between sexes although the general trend is an increasing incidence with advancing age. This pattern is shown in more detail in Fig. 1. The overall incidence in younger age groups was low e.g. 0.8 per 100,000 in those 25–29 years. The marked increase in incidence was seen from 60 years upwards (200.86 per 100,000). A peak was reached at 70–74 years where the rate was 349.06 per 100,000 population and following a fall in the 75–79 year group the rate increased once more before declining in the 85+ years age group. While women reached their peak incidence at 70–74 years (210.53 per 100,000), men did not reach theirs until 80–84 years (694.12 per 100,000). The male to female incidence ratio rose steadily in each decade of age from a minimum of 0 in those aged 20–29 years through each decade—0.5, 1.9, 2.3, 2.5, and 2.9, respectively—to a maximum of 4.1 in those aged 80+ years (Table 2).

**Table 2:** Regional incidence rates for lung cancer (per 100,000 population) by age and sex

Region/age	Males			Females		
	No.	Pop.	Rate	No.	Pop.	Rate
**NI**						
20-	0	124,358	0.0	1	123,984	0.8
30-	1	105,543	1.0	2	107,230	1.9
40-	19	93,128	20.4	10	94,699	10.6
50-	63	71,740	87.8	29	75,915	38.2
60-	228	61,212	372.5	106	73,067	145.1
70-	208	37,655	552.4	104	54,762	189.9
80+	82	12,394	661.6	47	29,294	160.4
Total	601	506,030	118.8	299	558,951	53.5
**E and W**						
20-	12	4178.9	0.3	5	4024.1	0.1
30-	90	3587.8	2.5	50	3532.1	1.4
40-	678	3416.7	19.8	397	3403.7	11.6
50-	2602	2636.0	98.7	1172	2648.7	44.3
60-	7924	2382.7	332.6	3770	2645.9	142.5
70-	9146	1531.3	597.3	4153	2167.4	191.6
80+	4297	576.6	745.2	2204	1376.0	160.2
Total	24,749	18,318.9	135.1	11,751	19,797.7	59.4
**Scotland**						
20-	2	417,522	0.5	0	406,000	0.0
30-	4	373,738	1.1	5	373,394	1.3
40-	97	329,420	29.4	66	334,196	19.7
50-	346	266,357	129.9	226	284,591	79.4
60-	1115	233,291	477.9	608	273,467	222.3
70-	1075	141,370	760.4	642	212,490	302.1
80+	471	50,148	939.2	306	125,062	244.7
Total	3,110	1,811,846	171.7	1853	2,009,200	92.2
**ROI**						
20-	1	279,211	0.4	2	271,451	0.7
30-	6	242,158	2.5	2	249,877	0.8
40-	26	224,107	11.7	21	221,346	9.5
50-	146	159,950	91.3	52	156,035	33.3
60-	318	124,861	254.7	128	134,718	95.0
70-	380	85,082	446.6	198	111,355	177.8
80+	109	28,091	388.0	65	49,212	132.1
Total	986	1,143,460	86.2	468	1,193,994	39.2

NB: Populations studied for ages 20+ years: NI = 1991/2, E and W = 1991, Scotland = 1992, and ROI = 1994

### Regional comparisons

- *(a) Overall Incidence*

Table 3 shows comparative incidence data (all ages and by sex) for the four major regions of the British Isles: Northern Ireland, England and Wales, Scotland and the Republic of Ireland. The data presented for the UK are the latest years available (at the time of writing) and 1994 is the first year for which incidence data were available on a national basis in the Republic of Ireland.

In the years studied, Northern Ireland had the lowest incidence of lung cancer within the UK. The highest rates were observed in Scotland where, remarkably, the crude rate was 1.7 times that observed in Northern Ireland, 1.4 times that observed in England and Wales and 2.4 times that seen in the Republic of Ireland. Among women the disparities were marginally greater: the crude incidence rate among Scottish women at 70.3 (per 100,000) was 2.7 times that of women in the Republic of Ireland. Allowing for age did not change the regional ranking but had the effect of reducing the differences between the regions, especially between Northern Ireland and England and Wales where the age-adjusted person rates were 38.3 and 39.0, respectively. The low age-standardised rates obtained in Table 3 result from the structure of the (younger) World reference population chosen to facilitate International comparisons. Overall, both comparisons indicated that the rates in the Republic of Ireland were the lowest in the British Isles. However, it should be noted that the pattern of incidence in the Republic differed fundamentally from that observed in the other regions as the incidence rate of 40.8 (per 100,000) was lower than the corresponding mortality rate of 43.3. Such a finding may be due, at least in part, to under-ascertainment.

**Table 3:** Summary of crude and adjusted* regional incidence rates per 100,000 population

Region	Males		Females		Persons	
	Crude	Adjusted	Crude	Adjusted	Crude	Adjusted
NI	78.2	59.7	37.0	22.5	57.0	38.3
E and W	99.0	60.2	45.0	22.9	71.4	39.0
Scotland	125.8	81.0	70.3	36.8	97.1	55.3
ROI	55.7	45.1	26.1	17.7	40.8	30.4

NB: Population years studied as Table 2, but for all ages. *World Population 1989

- *(b) Model Fitting*

In order to compare the incidence of lung cancer in the four regions more formally, we adopted the model fitting approach described above. Using a linear logistic model we analysed the incidence in relation to three factors: Age, Sex and Region. A number of different linear logistic models were fitted to the regional data.

The null hypothesis of a common relationship between Incidence, Age and Sex in all four regions was tested. The regional homogeneity hypothesis was found to be untenable ($\chi ^2$ = 850.4, df = 50, $p<<$ 0.01). This result suggests that the pattern of incidence by age and sex is rather complicated and that it may be more appropriate to fit models separately for males and females and for each region. Accordingly, the simple model:

2 $$\log_{e}[\theta /(1-\theta )] = \beta _{0} + \beta _{1}({\text{Age}}) + \beta _{2}({{\text{Age}}^{2}})$$

in which the logarithm of the odds on incidence is a quadratic function of age was used.

Table 4 summarises the results obtained. The quadratic model provided an adequate description of the incidence observed amongst: (a) Northern Irish males and females, (b) Scottish females and (c) Southern Irish males. Formally, it narrowly failed to describe the trend in age incidence in Southern Irish females ($\chi ^2$ = 10.4, df = 4, $p < 0.05$, but almost all of the contribution to $\chi ^2$ arose in one cell (20–29 yrs) in which 2 cases were observed, but only 0.27 were expected on the basis of the model. Ignoring this cell, the model also provides a satisfactory fit to the female data in the Republic.

**Table 4:** Linear logistic models in age and age-squared fitted to the regional incidence data

Region	Sex	$\hat{\beta _0}$	$\hat{\beta _1}$	$\hat{\beta _2}$	$\chi ^2$	*p*
NI	Male	-6.88 (0.08)	1.49 (0.09)	-0.30 (0.03)	7.5	>0.05
	Female	-7.60 (0.10)	1.23 (0.10)	-0.28 (0.04)	6.2	>0.05
E and W	Male	-6.87 (0.01)	1.36 (0.01)	-0.24 (0.004)	39.7	<0.05
	Female	-7.59 (0.02)	1.27 (0.02)	-0.29 (0.006)	44.3	<0.05
Scotland	Male	-6.56 (0.03)	1.42 (0.04)	-0.27 (0.01)	24.0	<0.05
	Female	-7.09 (0.04)	1.26 (0.05)	-0.30 (0.02)	4.2	>0.05
ROI	Male	-7.06 (0.05)	1.41 (0.06)	-0.30 (0.02)	6.0	>0.05
	Female	-7.88 (0.08)	1.22 (0.08)	-0.25 (0.03)	10.4	<0.05

Maximum Likelihood estimates of the parameters and their standard errors (in brackets)

NB: $\chi ^2$ has df = 4, model fits if *p* > 0.05, and age = (age-55)yrs

The regression coefficients in Table 4 are all well identified statistically and their quantitative similarity is worth noting, particularly in relation to the lung cancer incidence data on the island of Ireland as a whole. Despite this, the model based on Eq. (2) did not explain the pattern of age-sex specific incidence observed in England and Wales nor among Scottish males. The observed and expected numbers of incident cases of lung cancer are shown in Table 5 for all 4 regions.

It will be noted that among males in England and Wales and in Scotland the model consistently and significantly: (a) over-estimated the incidence among those aged 50–59 years, (b) under-estimated the incidence in the next decade and (c) over-estimated the incidence among men aged 70–79 yrs. This pattern of lack of fit is also evident to a lesser (non-significant) extent in the incidence data for Northern Ireland, but is completely absent from the data observed in the Republic of Ireland. For females in England and Wales the model again failed to fit the observed numbers of incident cases aged 50–69 years (Table 5). However, the pattern of lack of fit differed somewhat from the male data observed in E and W.

**Table 5:** Regional comparison

Region/Age	Males		Females	
	Observed	Expected	Observed	Expected
**NI**				
20-	0	0.10	1	1.13
30-	1	1.68	2	1.50
40-	19	16.04	10	10.44
50-	63	73.56	29	37.89
60-	228	205.73	106	94.71
70-	208	228.68	104	105.82
80+	82	75.20	47	48.49
**E and W**				
20-	12	8.76	5	3.19
30-	90	63.35	50	43.60
40-	678	717.64	397	362.24
50-	2602	2733.92	1172	1345.21
60-	7924	7605.58	3770	3529.96
70-	9146	9381.24	4153	4255.29
80+	4297	4238.51	2204	2191.50
**Scotland**				
20-	2	0.78	0	0.50
30-	4	10.72	5	7.36
40-	97	86.70	66	57.96
50-	346	376.60	226	236.56
60-	1115	1036.36	608	593.53
70-	1075	1155.53	642	656.65
80+	471	443.34	306	300.44
**ROI**				
20-	1	0.23	2	0.27
30-	6	3.72	2	2.97
40-	26	34.73	21	19.11
50-	146	137.31	52	59.01
60-	318	325.95	128	134.51
70-	380	371.23	198	176.99
80+	109	112.82	65	75.13

Observed and expected numbers based on the logistic model in age and age-squared by age, sex and region

NB:

1. Population years as in Table 3

2. Standardized residual, $z=({\mathrm {obs}}-{\mathrm {exp}})/\surd ( {\mathrm {exp}} )$ and $z^2 \approx \chi ^2$ with df=1

## Discussion

The data analysed above are now some 30 years old and this must be borne in mind when interpreting the findings above and the discussion which follows. Looking back, our main concern was that the absence of standard cancer registration facilities in Northern Ireland was limiting information about the true extent of lung cancer in the community. This deficiency had long concerned physicians, surgeons and epidemiologists alike. Accordingly, the study was undertaken to ascertain, for the first time, the incidence of lung cancer in Northern Ireland.

### Method

From the outset methodological considerations were paramount and a comprehensive multi-source notification design was adopted in order to maximise the ascertainment of cases of lung cancer. The procedures implemented in this study were even more comprehensive—especially with respect to the number of sources polled and the amount of pro-active searching— than those currently being followed by the recently established NI Cancer Registry. Accordingly, very few cases, if any, were thought to have been missed.

The findings, that no single source was sufficiently complete and that only 137 of the 900 incident cases were known to all 4 sources during the course of the study constitute a rather remarkable indictment of the reporting systems which existed in Northern Ireland during 1991/1992. Thus, Table 1 should prove a valuable aid to the workings of the new registry. However, it is unlikely that the method of notification of cancer of the lung is peculiarly unique and the need for the NI Registry to pro-actively pursue a comprehensive (multi-source) notification strategy was clear.

The number of incident cases, at 900, exceeded the mortality by 112 cases. As noted earlier mortality had been used consistently by the DHSS and other health planners as a surrogate measure for incidence in the Province. The results show that this particular strategy under-estimated the true extent of the disease in the community by 12.45%. In the absence of temporal incidence data it was impossible to estimate the duration of such under-estimation, but the implications for funding and resource allocation in the Province would not be lost on specialists in the field.

### Local comparisons

Although, in the years studied, Northern Ireland had the lowest incidence of lung cancer in UK the overall rate was still 40% higher than the rate in the Republic of Ireland which, on these data, had the lowest rate in the British Isles. The absence of national incidence data in the Republic for 1991–1992 hampers the interpretation of the comparison. As we remarked earlier, the data for 1994 were the first to become available on a national basis in the Republic and some allowance must be made for potential under-ascertainment in the early years of the development of a new registry. Even so, it is highly unlikely that any such allowance would alter the Republic’s ranking in the regional comparison.

The null hypothesis of regional homogeneity in the age-sex incidence distribution was rejected. Despite this, we have demonstrated that a simple sex specific model in which the logarithm of the odds on incidence is a quadratic function of age fitted the Northern Ireland and Republic of Ireland data adequately. This model also explained the age-specific incidence distribution among Scottish females. However, it did not fit the data for England and Wales nor for Scottish males: i.e., the log-odds on incidence does not follow a simple quadratic form in age. It may be, of course, that a more general model would apply in each region and we recognise that factors such as smoking status, exposure to passive smoke, and socio-economic status have not been taken into account in this paper.

### International comparisons

On a world-wide scale, the 1991–1992 Northern Ireland incidence rate of 57.0 per 100, 000 population was intermediate, being lower than some countries—England and Wales, Scotland, USA, and Canada—but, higher than others—Australia, India, Israel and Thailand [5–8]. Neither was the male to female incidence ratio of 2.1 to 1 particularly unusual. Data from the International Agency for Research on Cancer [3] showed marked inter-country variation in male to female incidence ratios, ranging from less than 3:1 (USA, Denmark and Israel) to those in excess of 9:1 (Germany, France and Italy). Accordingly, it seems that individuals in Northern Ireland were not at particularly high risk of developing the disease in 1991/92.

**Fig. 2:**
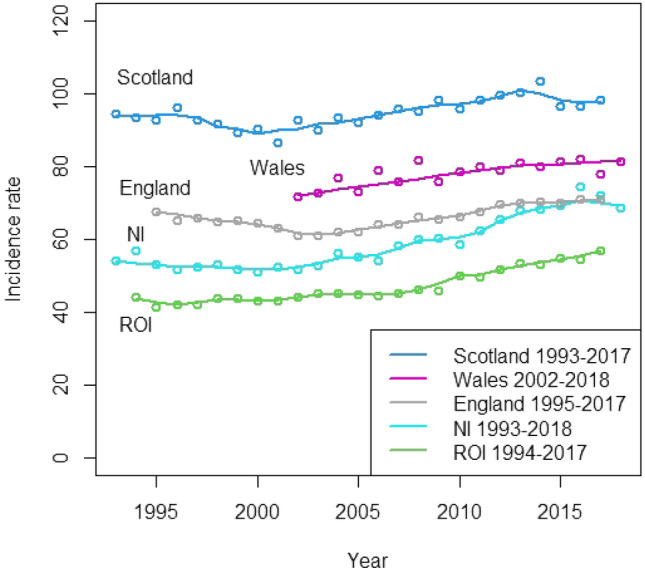
Trends (1993–2018) in crude incidence rates (persons per 100,000 population) for cancer of the lung by region with non-parametric smooth trend lines

### Postscript

In 2021, the epidemiological picture is rather different and the purpose of this new section is simply to bring the regional incidence results up to date.

Examining current Registry data [29–33], we were able to compile Fig. 2 which shows the regional trends in crude incidence rates for the period 1993–2018. Not all Registries have data available for the whole period: for example, the Welsh Registry has only published data from 2002.

The pattern which emerges is hardly re-assuring, all of the trends are increasing. The figure does, however, largely confirm the 1991–1992 ranking (highest–lowest) of Scotland, England, Northern Ireland and the Republic and confirms the conclusion that the position of ROI would remain unchanged, when its registry was fully operational.

It may be argued that the increasing trends are due to aging populations. Whilst this may be true in part, it seems an unlikely explanation for the trend in NI, where there was a shallow decline after 1991–1992 until c2000. Thereafter, a gradual increasing trend was experienced until 2010, when the increase accelerated up to the higher rates observed in 2016–2018. The 2017 rate in NI is c72, an increase of 26.3% over the 1991–1992 rate (57.0, Table 3) and it has been driven by a dramatic rise in incidence among females (not shown). The most striking finding is that the incidence rate in NI exceeded the rate in England in 2016! In 1991–1992 epidemiologists would not have thought such a result possible.

These increases in NI and elsewhere are the more puzzling, when considered against a backcloth of successful smoking bans in public places and declining population smoking prevalence rates in the UK, down from 20.2% in 2011 to 14.1% in 2019, [34], overall, and in the Republic, down from 28.3% in 2008 (last high) to 15.0% in 2020 (HSE, 2020) [35].

Thus, it seems that the conclusion reached about NI’s position in 1991–1992 was rather optimistic and further studies are now required to identify the factors underpinning these increasing trends.

## Footnotes

1 The Ulster Cancer Foundation, and the Department of Epidemiology and Public Health, Queen’s University of Belfast, actively encouraged the establishment of the NI Cancer Registry bringing the late Dr. Calum Muir, then Deputy Director of WHO, to Belfast as a distinguished international advisor.
